# Carotid plaque magnetic resonance imaging and recurrent stroke risk: A protocol for systematic review and meta-analysis

**DOI:** 10.1097/MD.0000000000015410

**Published:** 2019-05-03

**Authors:** Fengbin Deng, Xinyu Hao, Zhuoran Tang, Changping Mu, Kang Li, Huaqiang Li

**Affiliations:** a Chongqing Hospital of the University of Chinese Academy of Sciences; b The 3^rd^ Hospital/Acupuncture and Tuina School, Chengdu University of Traditional Chinese Medicine; c Big Data Research Center, China.

**Keywords:** carotid plaque, protocol, recurrent stroke, systematic review

## Abstract

**Background & Aims:**

Carotid atherosclerotic plaque is an important cause of carotid artery stenosis. The features of carotid atherosclerotic plaque detected by relevant magnetic resonance imaging (MRI), such as lipid core, plaque hemorrhage, and fibrous cap rupture, have been confirmed to be associated with the occurrence of the first cerebral ischemic event. Meanwhile, the features of carotid atherosclerotic plaque can be used as biomarkers to predict the occurrence of cerebral ischemic event. However, the mechanism of recurrent stroke is still unclear. A systematic review and meta-analysis will be performed to summarize the association between features of carotid artery plaque detected by MRI and recurrent stroke, so as to find biomarkers that can predict recurrent stroke.

**Methods:**

Electronic search will be performed in PUBMED, EMBASE, Cochrane Controlled Register of Trials (CENTRAL) from inception to October 30, 2018. We will include cohort studies with an average follow-up time of >1 month in which lipid-rich/necrotic cores (LRNC), intraplaque hemorrhage (IPH), and thinned or ruptured fibrous caps (TRFC) are associated with recurrent ipsilateral stroke or ischemic events. We will perform heterogeneity assessment before carrying out meta-analysis. According to the heterogeneity, we select random effect model or fixed effect model for meta-analysis of the included cohort studies.

**Results:**

Review Manager 5.3 software will be used to calculate the combined hazard ratio value and 95% confidence interval (CI). This meta-analysis will provide high-quality data analysis of LRNC, IPH, and TRFC and ipsilateral recurrent stroke or ischemic events, including biomarkers as major predictors.

**Conclusion:**

The systematic review will provide evidence to assess the association between features of carotid plaque and ipsilateral recurrent stroke or ischemic events.

**PROSPERO registration number:**

PROSPERO CRD42019124043.

## 1 Introduction

Previous studies have shown that the degree of carotid artery stenosis is a biological indicator to evaluate the occurrence of stroke events.^[1]^ Subsequent studies have demonstrated that the components of carotid atherosclerotic plaques that contribute to carotid artery stenosis can be well detected by magnetic resonance imaging (MRI) and also used as a tool for predicting and assessing stroke risk.^[2,3]^ Meanwhile, carotid endarterectomy (CEA), as a secondary intervention measure for stroke, plays an important role in preventing future stroke.^[4]^ However, the risk–benefit ratio of carotid endarterectomy for low-risk patients with carotid atherosclerotic plaque remains to be determined.^[5]^ Nevertheless, CEA treatment may be helpful for some patients with less severe carotid artery stenosis with the characteristics of plaque vulnerability.^[6]^ Therefore, whether patients with symptomatic carotid plaque need CEA to prevent recurrence of stroke events is very important for whether patients need to bear the risks and treatment costs brought by the surgery. The components of carotid artery plaque, such as thinned or ruptured fibrous caps (TRFC), lipid-rich/necrotic cores (LRNC), and intraplaque hemorrhage (IPH), etc., as biomarkers for predicting the first occurrence of stroke,^[7]^ whether they can continue to be used as tools for predicting and evaluating the risk of recurrence of stroke events needs to be further confirmed. However, recent studies have shown that IPH, TRFC, and LRNC can be used as biological indicators to predict recurrent cerebral ischemic events.^[5,8,9]^ Therefore, we plan to conduct a systematic review and meta-analysis to further confirm whether the components of carotid artery plaque can be used as an independent biological indicator to predict and evaluate the risk of stroke recurrence.

## 2 Methods

### 2.1 Study registration

The protocol has been registered in the International Prospective Register of Systematic Reviews (PROSPERO) with registration number CRD42019124043 on February 19, 2019.

### 2.2 Ethics and dissemination

The protocol of this systematic review will be disseminated in a peer reviewed journal and presented at relevant conferences. It is not necessary for a formal ethical approval, because the data are not individualized.

### 2.3 Inclusion criteria for study selection

#### 2.3.1 Types of studies

We will include cohort studies, with language limited to English. Specific inclusion criteria were: English language articles; MRI of carotid vessel plaque composition; mean follow-up >1 month; assessment for development of recurrent ipsilateral stroke or recurrent TIA; prospective cohort study. The exclusion criteria were: articles without specific data provided; case reports and reviews were excluded. This systematic review and meta-analysis is an evaluation study on the characteristic components of carotid artery plaque and the risk of recurrent stroke events, which does not involve the accuracy test of MRI sequence diagnosis or the study on the histopathology of carotid artery plaque. We included 3 specific plaque elements in this study: IPH; LRNC; and TRFC.

#### 2.3.2 Types of participants

Patients in the included study were adults and had prior cerebral ischemic symptoms, including hemispheric transient ischemic attack, amaurosis fugax, or non-disabling stroke, and were followed up for at least 1 month, all of which were confirmed by the study center, experienced physicians, or clinical consultants. Regardless of the recurrence of cerebral ischemic events, all patients received MRI scans of carotid artery plaques before or after the second intervention.

### 2.4 Search methods for the identification of studies

#### 2.4.1 Electronic searches

Electronic search will be performed in PUBMED, EMBASE, Cochrane Controlled Register of Trials (CENTRAL) from inception to Oct 30, 2018. We will use the Mesh terms and keywords in combination: carotid artery, carotid plaque, atherosclerosis, stroke, recurrent, recurrence, ischemic events. The search strategy for PubMed is shown in Table 1.

**Table 1:**
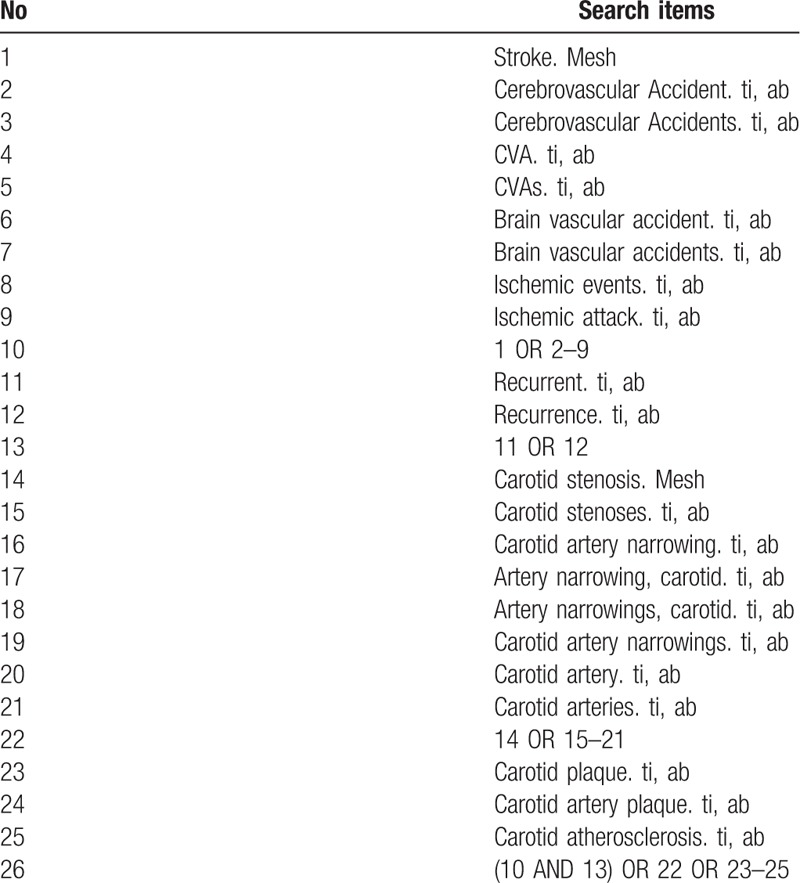
Search strategy used in PubMed.

#### 2.4.2 Searching other resources

The reference list of previously published systematic reviews focusing on predicting the risk of stroke and eligible studies will be searched. We will manually retrieve conference proceedings and academic exchange summaries.

### 2.5 Data collection and analysis

#### 2.5.1 Selection of studies

**Figure 1:**
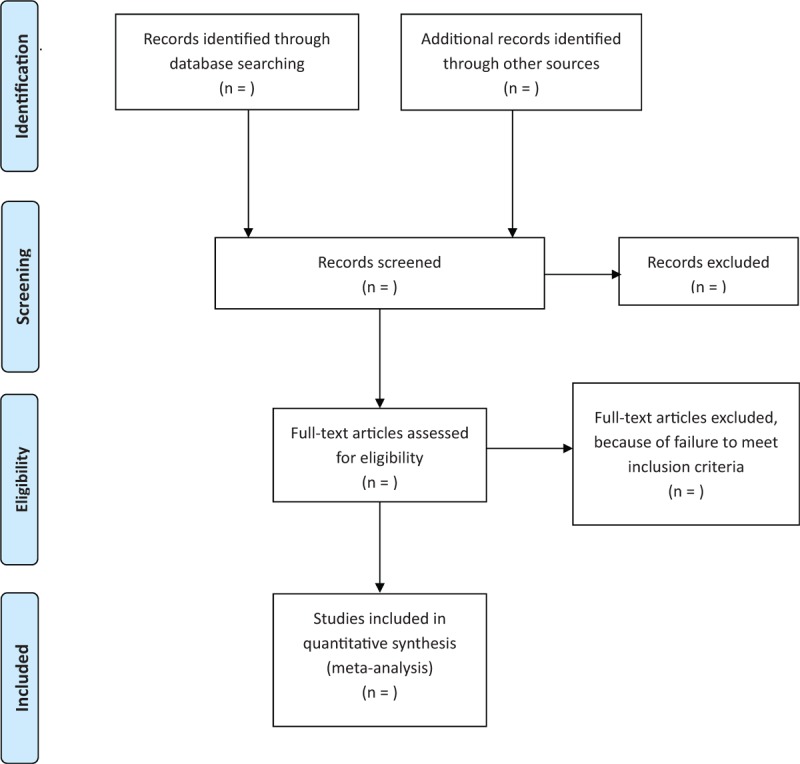
Flow diagram of study selection process.

Before the search begins, each reviewer will receive professional training to ensure consistency in the selection process and avoid the risk of bias (ROB) in human factors. The screening process will use EndNote X8 literature management software. The 2 review authors will read the topics, abstracts, keywords, and the full text independently if needed. The screening process will be strictly in accordance with the inclusion criteria. If there is a disagreement, it will be decided with another reviewer. We will record the exclusion reason for the excluded literature. The details of selection process will be shown in the Preferred Reporting Items for Systematic Reviews and Meta-Analyses (PRISMA) flow chart (Fig. 1).

#### 2.5.2 Data extraction and management

Two reviewers will extract information from eligible studies using a predefined data extraction form. We will include the needed information via 4 parts:

Characteristics of the included trials: the first author, year of publication, study design, total sample size; characteristics of participants: age (mean age), sex (male, %), number of medically managed participants, number of carotid arteries with follow-up outcome data, disease severity (mean degree of arterial stenosis), hypertension, diabetes, smoking, diabetes mellitus, type of symptom on presentation, ipsilateral recurrent strokes in negative test group, ipsilateral recurrent strokes in positive test group, time from presenting symptom and MRI, the mean time interval between the second MR scan and the recurrent event, number of arteries with negative MRI, number of arteries with positive MRI, ipsilateral all ischemic events in negative or positive test group; characteristics of MRI scan: parameters of MRI scan, scan sequence; characteristics of outcome: imaging of plaque elements, all ischemic event HR, 95% CI.

### 2.6 Risk of bias assessment

We plan to use the Newcastle-Ottawa Scale for assessing risk of bias which mainly includes 4 specific domains: trials population selection, comparability between groups, the results of evaluation or evaluation of exposure factors. Methodological quality will be considered as low risk, high risk, or unclear risk of bias. Two reviewers will complete the easement of risk of bias, disputes shall be settled through discussion, or a third party shall participate in judgment and arbitration.

### 2.7 Data synthesis

RevMan 5.3 (Review Manager (RevMan) [Computer program]. Version 5.3. Copenhagen: The Nordic Cochrane Centre, The Cochrane Collaboration, 2014) software provided by Cochrane collaboration (www.cochrane.org) will be used to conduct meta-analysis and synthesis. The HR, a potentially more useful measure of risk taking into account time to events, can be more significant for our systematic meta-analysis. The HR that demonstrating relationship between IPH, LRNC, and TRFC and the risk of recurrent ischemic events (Stroke plus TIA) and 95% confidence interval (95% CI) will be detected, respectively, for each specific plaque element.

### 2.8 Quality of evidence assessment

The Grading of Recommendations Assessment, Development, and Evaluation (GRADE) will be used as a tool for assessing the strength of the body of evidence. According to the GRADE evidence evaluation system, the level strength of evidence can be divided into the following 4 levels: high, moderate, low, or very low.

### 2.9 Heterogeneity assessment

Heterogeneity between studies will be evaluated by using the Cochrane *Q* test (*P* < 0.10 will be defined as statistically significant heterogeneity), and it will be quantified with *I*^2^ statistics (heterogeneity will be considered as low, moderate, large, and very large for *I*^2^ <25%, 25%–49%, 50%–74%, and ≥75%, respectively). When there is very large heterogeneity between studies, we will run meta-regression to locate the source of heterogeneity and perform subgroup analysis.

### 2.10 Assessment of reporting bias

Egger test will be used to assess the publication bias and the small study biases. When a *P*-value <.10 in the regression asymmetry is found, we will consider the existence of small-study effect.

### 2.11 Meta-analysis

Meta-analysis will be performed by using RevMan (Review Manager, version 5.3). The HRs of the included studies will be combined by using both fixed-effects model and random-effects model, and we will check the consistency of the results between the 2 models. When *I*^2^ <50%, we will report the result generated by the fixed-effects model; otherwise, we will choose the result by the random-effects model.

### 2.12 Sensitivity analysis

We will perform the following sensitivity analyses: excluding studies with high or unclear risk of bias; excluding studies with total sample size <50 participants; excluding studies with significant small-study effect.

### 2.13 Meta-regression and subgroup analysis

If there is a significant heterogeneity in the included trials, subgroup analysis based on treatment, parameters of MRI scan, stenosis degree, and other potential factors will be performed to explore the possible factors of heterogeneity.

### 2.14 Publication bias

Egger test will be used to assess the publication bias and the small study bias. When a *P*-value <.10 in the regression asymmetry is found, we will consider the existence of small-study effect. However, if there are <10 articles included, publication bias will not be explored.

### 2.15 Trial sequential analysis

Meta-analysis may generate false-positive findings (type I error) because of repeated significance testing. To assess the risk of type I error, we will apply trial sequential analysis (TSA), which estimates the information size (cumulated sample size of the included studies) and adjusts threshold for statistical significance (TSA software, version 0.9 beta).

## 3 Discussion

The main components of carotid artery plaque are IPH, LRNC, TRFC.^[3]^ A large number of histopathological studies and prospective studies have confirmed that IPH, LRNC, and TRFC are unstable factors of the arterial plaque and stimulate the plaque progression.^[10,11]^ At the same time, these plaque components can be evaluated noninvasively by MRI.^[7,12–16]^ MRI features of carotid artery plaques can assess the risk of ipsilateral ischemic events, which has been confirmed by a systematic review and meta-analysis.^[17]^ Patients with symptomatic severe carotid artery stenosis have a decreased risk of recurrence of cerebral ischemic events after CEA surgery.^[18]^ However, the using of carotid stenosis to assess carotid endarterectomy is considered imperfect, which determining the risk of recurrence in some patients remains difficult.^[19,20]^ Recent studies have shown that carotid artery plaque components detected by Magnetic resonance technique can be used not only as biomarkers to assess the risk of ipsilateral stroke, but also to assess the risk of recurrent stroke or recurrent ischemic events. This provides evidence for defining the risk of recurrent stroke or ischemic events in patients with symptomatic carotid plaque. To our knowledge, there is no related systematic review evaluating the association between carotid plaque characteristics and recurrent stroke or recurrent ischemic events. Therefore, we will conduct a systematic review and meta-analysis to assess the association between MRI features of carotid artery plaques and recurrent stroke or recurrent ischemic events. Our aim is to provide reliable evidence to help clinicians make decisions selecting patient selection for CEA surgery.

## Author contributions

Fengbin Deng, Kang Li, and Xinyu Hao conceived the systematic review. All the authors helped to write the article. Fengbin Deng, Kang Li, and Huaqiang Li were responsible for the protocol and the statistical analysis plans. Fengbin Deng and Zhuoran Tang will contribute to the original data acquisition. All the authors approved the publication of the protocol.

**Conceptualization:** Kang Li, Huaqiang Li.

**Data curation:** Fengbin Deng, Xinyu Hao.

**Data extraction:** Fengbin Deng, Xinyu Hao.

**Data analysis**: Fengbin Deng, Xinyu Hao.

**Formal analysis:** Fengbin Deng, Xinyu Hao.

**Investigation:** Fengbin Deng.

**Methodology:** Fengbin Deng, Xinyu Hao.

**Software:** Zhuoran Tang, Changping Mu, Fengbin Deng.

**Supervision:** Kang Li, Huaqiang Li.

**Writing – original draft:** Fengbin Deng, Xinyu Hao.

**Writing – review & editing:** Fengbin Deng, Xinyu Hao.

Kang Li orcid: 0000-0003-4637-6620.

Fengbin Deng orcid: 0000-0003-0510-0839.
